# Functional characterization of the α_1_-adrenoceptor in adult male rat locus coeruleus neurons *ex vivo*

**DOI:** 10.3389/fphar.2025.1626019

**Published:** 2025-08-21

**Authors:** Irati Rodilla, Aitziber Mendiguren, Joseba Pineda

**Affiliations:** Department of Pharmacology, Faculty of Medicine and Nursing, University of the Basque Country (UPV/EHU), Leioa, Bizkaia, Spain

**Keywords:** locus coeruleus, α_1_-adrenoceptor, slice, firing, noradrenaline, rat, cirazoline, phenylephrine

## Abstract

**Introduction:**

The α_1_-adrenoceptor (α_1_AR) is involved in the physiopathology of the central nervous system (CNS), but its function in the adult male rat locus coeruleus (LC) has not been fully studied. We aimed to characterize the role of the α_1_AR in the regulation of the firing rate (FR) of LC neurons and to describe the signaling pathways involved.

**Methods:**

We measured, through single-unit extracellular recordings of LC neurons from adult male rats were used to measure the effect of adrenergic agonists in the presence and absence of adrenergic antagonists or inhibitors of several signalling pathways.

**Results:**

Noradrenaline (NA) (100 µM) and phenylephrine (PE) (100 µM) induced a stimulatory effect in the presence of α_2_-adrenoceptor (α_2_AR) antagonist RS 79948 (0.1 µM). The α_1_AR agonist cirazoline (1–100 µM) also stimulated the FR of LC neurons. The stimulatory effects of NA (100 µM), PE (100 µM), and cirazoline (1 μM and 10 µM) were blocked by α_1_AR antagonist WB 4101 (0.5 µM). NA (100 µM)-induced stimulation was reduced in the presence of G_i/o_ protein inactivator pertussis toxin (PTX) (500 ng ml^-1^) and the transient receptor potential (TRP) channel blocker 2-APB (30 µM), but not by protein kinase C (PKC) inhibitor Go 6976 (1 µM), G protein-activated inward rectifier potassium (GIRK) channel blocker BaCl_2_ (300 µM), or protein kinase A (PKA) inhibitor H-89 (10 µM). The stimulatory effect of cirazoline was not reduced by any of the tested inhibitors.

**Conclusions:**

From α_1_AR activation stimulates the FR of adult rat LC neurons through a signaling pathway that involves G_i/o_ proteins and TRP channels.

## 1 Introduction

The α_1_-adrenoceptor (α_1_AR) and the α_2_-adrenoceptor (α_2_AR), which belong to the G protein-coupled receptor (GPCR) family, have been involved in brain developmental processes and constitute potential therapeutic targets for different neuropathological disorders such as drug addiction, Parkinson’s and Alzheimer’s diseases, or post-traumatic stress (Ghanemi and Hu, 2015; Perez, 2020). The α_1_AR predominantly couples to G_q/11_, the stimulation of which leads to the activation of phospholipase C (PLC) and the production of inositol 1,4,5-trisphosphate (IP_3_) and diacylglycerol (DAG). IP_3_ activates the release of Ca^2+^ into the cytoplasm, whereas DAG activates protein kinase C (PKC) (Hein and Michel, 2007). The α_2_AR couples to the G_i/o_ protein, the activation of which results in the inhibition of adenylyl cyclase and reduction of cyclic adenosine monophosphate (cAMP) and protein kinase A (PKA) activity (Aantaa et al., 1995). However, each adrenoceptor can couple to multiple signaling pathways. Thus, the α_1_AR can activate G_i_ proteins (Petitcolin et al., 2001) and the α_2_AR can couple to G_s_ proteins (Eason and Liggett, 1995). Furthermore, adrenergic agonists can display ligand-directed signaling bias (Alexander et al., 2023).

Locus coeruleus (LC) is the main noradrenergic nucleus in the central nervous system (CNS) (Foote et al., 1983). It is involved in the regulation of CNS functions, including sleep–wake cycle, attention, memory, and stress-related responses (Berridge and Waterhouse, 2003; Matt et al., 2024). The activity of LC cells is regulated, among others, by the αAR (Schwarz et al., 2015). Both α_1_AR and α_2_AR have been localized in LC neurons through quantitative autoradiography (Chamba et al., 1991) and RT-PCR (Osborne et al., 2002). *In situ* hybridization techniques have revealed that the α_1A_AR is the main subtype of the α_1_AR in the LC (Day et al., 1997). Moreover, a recent immunohistochemical study has shown that the α_1_AR colocalizes with tyrosine hydroxylase in LC dendrites, which indicates that the α_1_AR is expressed in NA neurons (Luyo et al., 2023). However, there are conflicting data regarding the functional role of the α_1_AR in LC neurons. Some authors have suggested that the α_1_AR decreases its activity during development and disappears in the adult male rat LC (Finlayson and Marshall, 1984; Williams and Marshall, 1987). In contrast, indirect evidence has suggested that the α_1_AR contributes to the excitability of LC neurons observed in the presence of the α_2_AR antagonist in adult male rat brain slices (Ivanov and Aston-Jones, 1995). Furthermore, the activation of the α_1_AR reduces outward potassium currents induced by α_2_AR activation in LC neurons (Osborne et al., 2002). Finally, microdialysis studies have shown that local administration of α_1_AR agonist cirazoline increases noradrenaline (NA) in the LC, whereas administration of an α_1_AR antagonist decreases it (Pudovkina et al., 2001; Pudovkina and Westerink, 2005).

Although the function of the α_1_AR in different brain areas of the CNS and its involvement in several neurological disorders has been studied (Ghanemi and Hu, 2015; Lemmens et al., 2015; Perez, 2020), the role of this receptor in the adult LC remains controversial. Therefore, the aim of this work was to characterize functionally the α_1_AR in LC neurons from adult male rats and to examine possible downstream processes linked to receptor activation through single-unit extracellular recordings in brain slices.

## 2 Materials and methods

### 2.1 Animals

A total of 99 adult male Sprague–Dawley rats (200–300 g) were used to perform electrophysiological assays. Animals were obtained from the animal facilities of the University of the Basque Country (Leioa, Spain) and housed (2–5 rats/cage) under controlled environmental conditions (22°C, 12:12 h light/dark cycles with the light phase starting at 8:00 a.m. and humidity of 65%–70%) with free access to food and water. All the experiments were carried out according to EU Directive 2010/63 on the protection of animals used for scientific purposes and reviewed and approved by the local Ethical Committee for Research and Teaching of the University of the Basque Country (UPV/EHU, Spain) and the Department of Sustainability and Natural Environment of Provincial Council from Bizkaia. All the efforts were made to minimize animal suffering and to reduce the number of animals used.

### 2.2 Brain slice preparation

Experiments were performed as previously described (Nazabal et al., 2023). Animals were anesthetized with chloral hydrate (400 mg kg^-1^, i.p.) and decapitated. The brain was rapidly removed, and a block of tissue containing the brain stem was immersed in ice-cold modified artificial cerebrospinal fluid (aCSF), where NaCl was equiosmotically substituted with sucrose to improve neuronal viability. Coronal slices of approximately 600 μM thickness containing the LC were cut using a vibratome (FHC Inc., Brunswick, GA, USA) and then allowed to recover from the slicing for 90 min in oxygenated aCSF (95% O_2_/5% CO_2_, pH = 7.34). Then, slices were placed on a nylon mesh in a modified Haas-type interface chamber continuously perfused with aCSF at a temperature of 33 °C and a flow rate of 1.5 mL min^-1^, as previously made in different studies performed in rat brain slices from the LC (Nazabal et al., 2023). This temperature maintains a balance between near-natural physiological functionality and adequate tissue viability suitable for stable recordings. The aCSF had the following composition: NaCl 130 mM, KCl 3 mM, NaH_2_PO_4_ 1.25 mM, D-glucose 10 mM, NaHCO_3_ 20 mM, CaCl_2_ 2 mM, and MgSO_4_ 2 mM. The modified aCSF, in which NaCl was equiosmotically replaced by sucrose, had the following composition: KCl 3 mM, NaH_2_PO_4_ 1.25 mM, D-glucose 10 mM, NaHCO_3_ 24 mM, sucrose 252 mM, CaCl_2_ 2 mM, and MgSO_4_ 2 mM.

### 2.3 Extracellular recordings

Single-unit extracellular recordings of LC noradrenergic neurons were made as previously described (Nazabal et al., 2023). The recording electrode consisted of an Omegadot glass micropipette (Sutter Instruments, Novato, CA, USA) pulled and filled with 50 mM NaCl, with the tip broken back to a size of 2–5 μm (3–5 MΩ). The electrode was placed in the LC, which was identified visually in the rostral pons as a dark oval area on the lateral borders of the central grey and the fourth ventricle, just anterior to the genu of the facial nerve. The extracellular signal recorded using the microelectrode was passed through a high-input impedance amplifier system (Axoclamp 2A, Axon Instruments, Foster City, CA) and monitored using an oscilloscope with an audio analyzer (Cibertec S.A., Madrid, Spain). Individual (single-unit) neuronal spikes were isolated from the background noise with a window discriminator and counted. FR was represented and analyzed using a PC-based custom-made program (HFCP^®^; Cibertec S.A. Madrid, Spain), which generated histogram bars representing the cumulative number of spikes in consecutive 10-s bins. Noradrenergic neurons in the LC were identified by the following electrophysiological criteria: a spontaneous and regular discharge, a slow firing rate (FR), and a positive–negative biphasic waveform of 3–4 ms duration (Andrade and Aghajanian, 1984). We only selected cells that showed stable firing rates between 0.5 and 1.5 Hz for at least 3–5 min and clear inhibitory responses to perfusion with [Met]enkephalin (ME, 0.8 μM, 1 min) or γ-aminobutyric acid (GABA, 1 mM, 1 min) according to previous studies (Nazabal et al., 2023).

### 2.4 Pharmacological procedures

To characterize the functional role of α_1_AR in LC neurons, first, the effect of nonselective AR agonist NA (100 μM, 1 min) was studied before or during the administration of α_2_AR antagonist RS 79948 (0.1 μM, 10 min). Next, α_1_AR agonists cirazoline (1, 10, 100 μM, 5–10 min) or phenylephrine (100 μM, 1 min) were perfused in the presence or absence of α_1_AR antagonist WB 4101 (0.5 μM, 10 min) to confirm that their effects on the FR were mediated by α_1_AR activation. To characterize the putative signaling pathways involved in the effects of α_1_AR agonists on the spontaneous FR of LC neurons, NA (100 μM, 1 min) or cirazoline (10 μM, 5–10 min) was tested during perfusion with the following drugs: Go 6976 (1 μM, 30 min; inhibitor of classical type PKC isoenzymes), chelerythrine (10 μM, 30 min; PKC inhibitor), U73122 (10 μM, 30 min; PLC inhibitor), H-89 (10 μM, 20 min; PKA inhibitor), BaCl_2_ [300 μM, 15 min; G protein-activated inward rectifier potassium (GIRK) channel inhibitor], or 2-APB (3, 10, and 30 μM, 10 min; blocker of TRPC5 and TRPM7 channels). Control applications of α_1_AR agonists were performed in the presence of the vehicle used to dissolve each inhibitor or blocker. To study the role of G_i/o_ proteins in the stimulatory effect induced by NA (100 μM, 1 min) or cirazoline (10 μM, 5–10 min), we treated brain slices with the catalyst of ADP-ribosylation of G_i/o_ protein pertussis toxin (PTX) (500 ng ml^-1^, 18 h). In PTX-pretreated cells, the effect of NA was tested in the same neuron both in the absence and presence of RS 79948 (0.1 μM, 10 min), whereas the effect of cirazoline was studied in different neurons because its effect was not washable. At the beginning of all the experiments, inhibitory effects of GABA (1 mM, 1 min) and ME (0.8 μM, 1 min) were measured to verify that slices were correctly perfused (inhibition magnitudes >80% of the basal firing rate). In PTX-pretreated cells, only neurons with a reduced inhibitory effect of ME (<80%) were considered.

### 2.5 Data analysis and statistics

The effects of AR agonists were calculated by subtracting the FR before drug perfusion from the FR after drug perfusion (FR_after_−FR_before)_. For the inhibitory effect of NA, FR_after_ was considered the average FR recorded for 60 s after agonist perfusion. For the stimulatory effect, FR_after_ was the average peak FR recorded for 30 s after perfusion with NA in the presence of RS 79948 or the average FR recorded for the last 120 s after perfusion with cirazoline. FR_before_ was the average FR recorded for 60 s before agonist administration. Effects were normalized as the percentage change from the baseline FR before the application of adrenergic agonist. The data and statistical analysis were carried out using the computer program GraphPad Prism (version 5.0 for Windows; GraphPad Software, Inc., San Diego, CA, USA) and comply with the recommendations on experimental design and analysis in pharmacology (Curtis et al., 2015). Values are expressed as the mean ± SEM of *n* experiments. The FR before or after drug application or the effects of drugs on the FR (% change from the baseline FR) were compared using a paired Student’s t-test within the same cell or an unpaired Student’s t-test between different groups. Comparisons between the effects of different concentrations of a drug in the same cell (cirazoline and 2-APB) were performed using the repeated-measures ANOVA, followed by Bonferroni’s multiple comparison *post hoc* test. Comparisons between the effect of cirazoline in the absence and presence of different inhibitors were performed using the one-way ANOVA, followed by the Dunnet *post hoc* test. The threshold of significance was considered as *P* = 0.05.

### 2.6 Drugs and reagents

The following drugs were purchased from Tocris Bioscience (Bristol, United Kingdom): 2-APB, chelerythrine chloride, cirazoline hydrochloride, Go 6976, H-89 dihydrochloride, PTX, PE hydrochloride, RS 79948 hydrochloride, and U73122. The following drugs were obtained from Sigma-Aldrich Química S.L. (Madrid, Spain): BaCl_2_ dihydrate, GABA, L-(−)-NA (+)-bitartrate salt monohydrate, and WB 4101 hydrochloride. ME acetate salt was purchased from Bachem (Weil am Rhein, Germany). Stock solutions of cirazoline, GABA, H-89, NA, PE, PTX, RS 79948, and WB4101 were first prepared in Mili-Q water and then diluted in aCSF to the final concentration before application. Go 6976, U73122, and 2-APB stock solutions were prepared in DMSO. The final concentration of DMSO was 0.2% (U73122 dilution), 0.03% (2-APB dilution), or 0.01% (Go 6976 dilution), which fail to change the FR of LC cells. BaCl_2_ was directly dissolved in aCSF.

## 3 Results

### 3.1 Effect of nonselective αAR NA and α_1_AR agonists PE and cirazoline on the firing rate of LC cells

**FIGURE 1:**
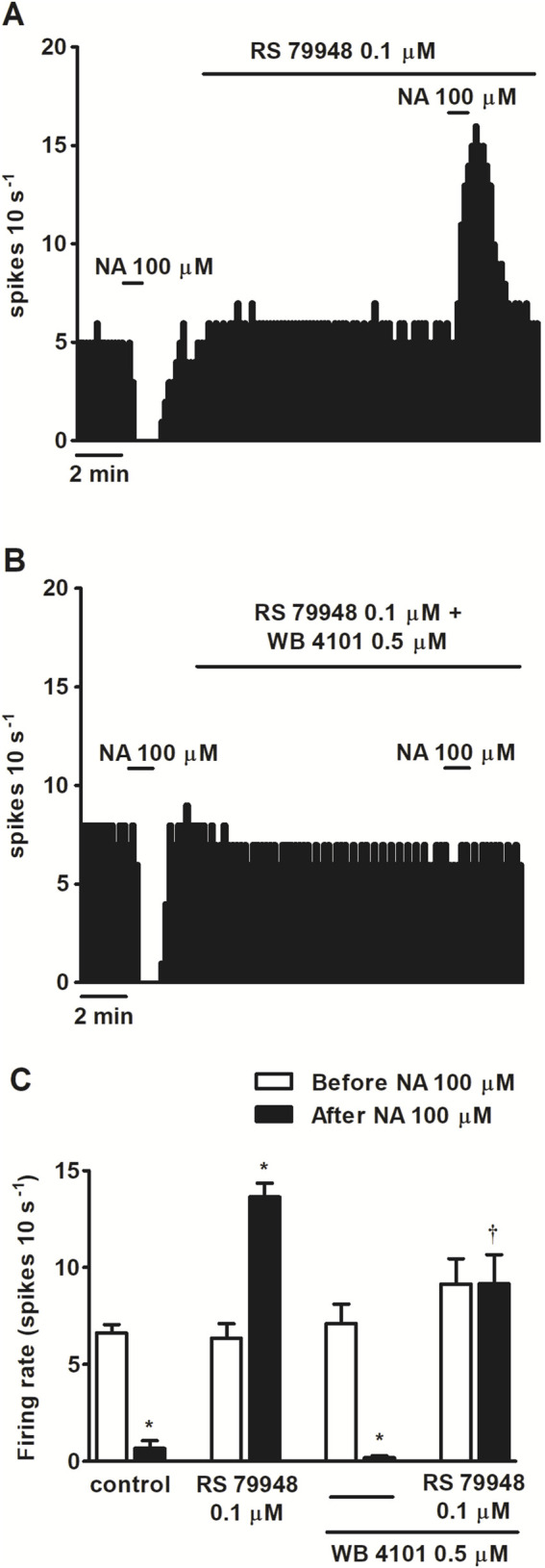
Effect of nonselective adrenergic agonist NA before and after the administration of α_2_AR antagonist RS 79948 or RS 79948 and α_1_AR antagonist WB 4101 on the spontaneous FR of LC neurons. **(A,B)** Representative examples of recordings of single LC neurons showing the inhibitory effect of NA (100 μM, 1 min) or its stimulatory effect in the presence of α_2_AR antagonist RS 79948 (0.1 μM, 10 min) on the basal FR **(A)** and the blockade of NA-induced effects in the presence of both RS 79948 (0.1 μM, 10 min) and α_1_AR antagonist WB 4101 (0.5 μM, 10 min) **(B)**. Vertical lines represent the integrated firing rates (spikes per 10 s). Drugs were bath-applied at the concentrations and for the durations indicated by horizontal bars. **(C)** Bar histograms showing the mean ± SEM of LC neurons FR before and after the application of NA (100 μM, 1 min, *n* = 5), NA (100 μM, 1 min) + RS 79948 (0.1 μM, 10 min, *n* = 5), NA (100 μM, 1 min) + WB 4101 (0.5 μM, 10 min, *n* = 6), or NA (100 μM, 1 min) + RS 79948 (0.1 μM, 10 min) + WB 4101 (0.5 μM, 10 min, *n* = 5). *P < 0.05, compared with the FR before the application of NA (100 μM, 1 min) using a paired Student’s t-test. †*P* < 0.05, compared with the effect (normalized as the percentage change from the baseline FR) induced by NA (100 μM, 1 min) in the absence of WB 4101 (0.5 μM, 10 min) during RS 79948 (0.1 μM, 10 min) perfusion using an unpaired Student’s t-test.

**FIGURE 2:**
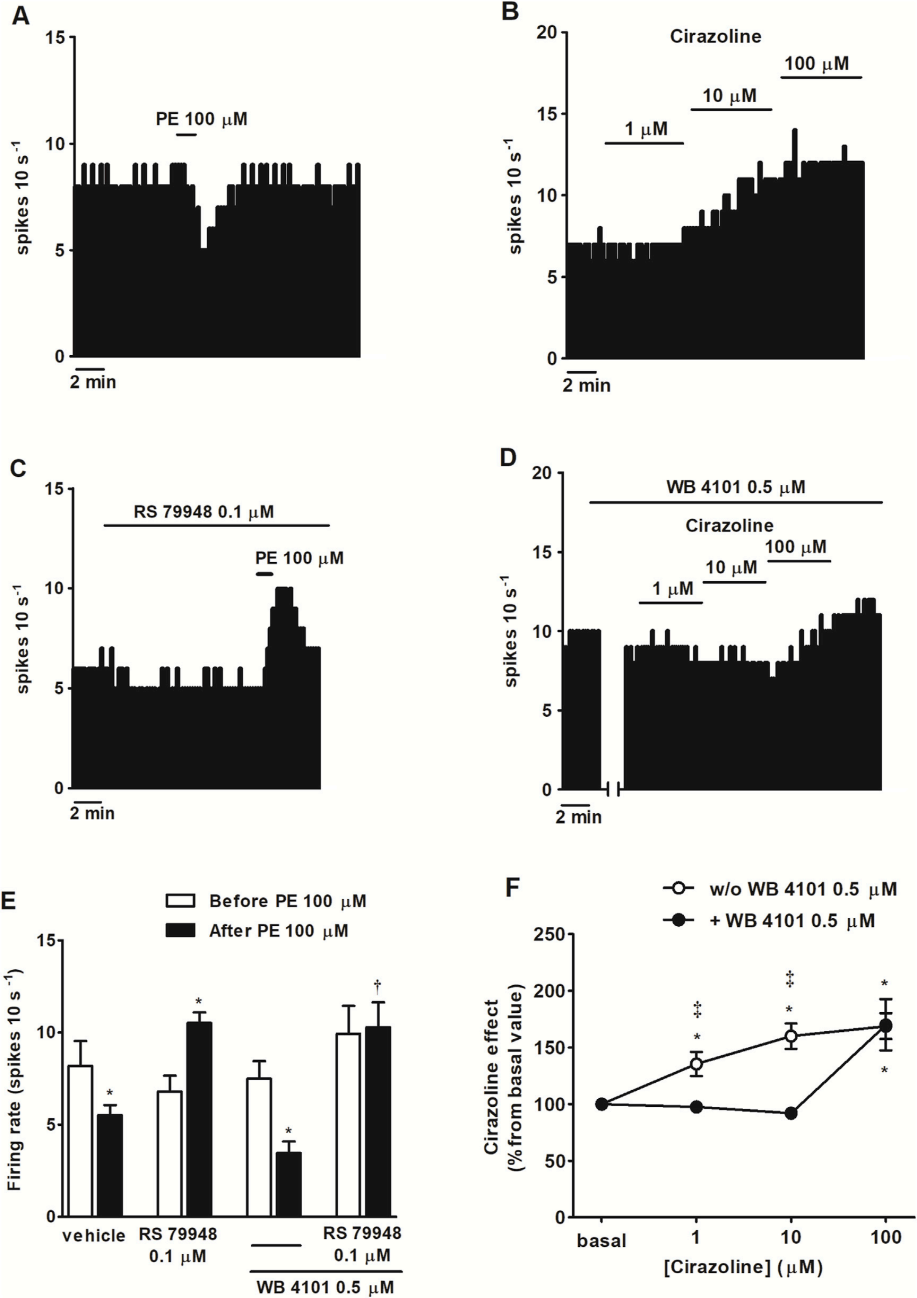
Effect of α_1_AR agonists PE and cirazoline before and after the administration of α_2_AR antagonist RS 79948, α_1_AR antagonist WB 4101 or RS 79948 and WB 4101 on the spontaneous FR of LC neurons. **(A–D)** Representative examples of recordings of single LC neurons showing the inhibitory effect of PE (100 μM, 1 min) on the basal FR **(A)**, the stimulatory effect of cirazoline (1, 10, and 100 μM; 5–10 min) **(B)**, the stimulatory effect of PE (100 μM, 1 min) in the presence of α_2_AR antagonist RS 79948 (0.1 μM, 10 min) **(C)**, and the blockade of the stimulatory effect of cirazoline (1, 10 μM, 5–10 min), but not cirazoline (100 μM, 5–10 min), in the presence of α_1_AR antagonist WB 4101 (0.5 μM, 10 min) **(D)**. Vertical lines represent the integrated firing rates (spikes per 10 s). Drugs were bath-applied at the concentrations and for the durations indicated by horizontal bars. **(E)** Bar histograms showing the mean ± SEM of LC neurons FR before and after the application of PE (100 μM, 1 min, *n* = 5), PE (100 μM, 1 min) + RS 79948 (0.1 μM, 10 min, *n* = 5), PE (100 μM, 1 min) + WB 4101 (0.5 μM, 10 min, *n* = 7), or PE (100 μM, 1 min) + RS 79948 (0.1 μM, 10 min) + WB 4101 (0.5 μM, 10 min, *n* = 5). **(F)** Symbols representing the mean ± SEM of LC neurons FR before and after the application of cirazoline (1, 10, and 100 μM; 5–10 min; *n* = 12) or cirazoline (1, 10, and 100 μM; 5–10 min) + WB 4101 (0.5 μM, 10 min, *n* = 5). *P < 0.05, compared with the FR before the application of PE (100 μM, 1 min) using a paired Student’s t-test or with the FR before the application of cirazoline (1, 10, and 100 μM, 5–10 min) using a repeated-measures ANOVA, followed by Bonferroni’s multiple-comparison *post hoc* test. †*P* < 0.05, compared with the effect (normalized as the percentage change from the baseline FR) induced by PE (100 μM, 1 min) in the absence of WB 4101 (0.5 μM, 10 min) during RS 79948 (0.1 μM, 10 min) perfusion using an unpaired Student’s t-test. ‡*P* < 0.05, compared with the effect of cirazoline in the presence of WB 4101 (0.5 μM, 10 min) using an unpaired Student’s t-test.

To study the functional role of α_1_AR in LC neurons, we tested the effects of several α-adrenergic agonists in the presence or absence of α-adrenergic antagonists on the spontaneous FR of LC neurons. As expected, application of the nonselective α-adrenergic agonist NA (100 μM, 1 min), which also binds to the β-adrenoceptor (βAR) and dopamine D_2_ receptor (D2 receptor), inhibited the FR of LC neurons by 90.5% ± 5.1% (*n* = 5, *P* < 0.05). However, in the presence of α_2_AR antagonist RS 79948 (0.1 μM, 10 min), NA (100 μM, 1 min) stimulated the FR by 114.3% ± 23.7% (*n* = 5, *P* < 0.05) (Figures 1A,C). After the application of α_1_AR antagonist WB 4101 (0.5 μM, 10 min), NA (100 μM, 1 min) induced a decrease of 98.8% ± 1.0% on the FR (*n* = 6, *P* < 0.05) (Figure 1C), whereas in the presence of both α_1_AR and α_2_AR antagonists, NA (100 μM, 1 min) failed to induce any significant change in the FR (*n* = 5) (Figures 1B,C). To study whether other drugs mimicked the α_1_AR-mediated effect of NA, we studied the effects of α_1_AR agonists PE (100 μM, 1 min) and cirazoline (1, 10, 100 μM, 5–10 min) on the FR of LC neurons. Perfusion with the α_1_AR agonist PE (100 μM, 1 min), which also shows affinity for α_2_AR and β_1_AR, inhibited the FR of LC neurons by 28.7% ± 7.6% (*n* = 5, *P* < 0.05) (Figures 2A,E). In the presence of RS 79948 (0.1 μM, 10 min), PE (100 μM, 1 min) stimulated the FR by 60.7% ± 12.4% (*n* = 5, *P* < 0.05) (Figure 2C), whereas in the presence of WB 4101 (0.5 μM, 10 min), it induced an inhibition of 52.9% ± 9.0% on the FR (*n* = 7, *P* < 0.05) (Figure 2E). The simultaneous application of RS 79948 (0.1 μM, 10 min) and WB 4101 (0.5 μM, 10 min) blocked both the inhibitory and stimulatory effects of PE (100 μM, 1 min) (*n* = 5) (Figure 2E). Finally, the administration of α_1_AR agonist cirazoline (1, 10, and 100 μM, 5–10 min), which also binds to the serotonin 1A receptor (5-HT_1A_ receptor) and α_2_AR receptors, induced a concentration-dependent increase in the FR of LC neurons. Thus, the stimulatory effect induced by cirazoline 1 μM was 35.5% ± 10.6% (*n* = 12, *P* < 0.05), whereas those induced by cirazoline 10 μM and 100 μM were 60.1% ± 11.2% (*n* = 12, *P* < 0.05) and 68.9% ± 11.5% (*n* = 12, *P* < 0.05), respectively (Figures 2B,F). In the presence of WB 4101 (0.5 μM, 10 min), which shows lower affinity for the D_2_ receptor and α_2_AR, the lowest concentrations of cirazoline (1 μM and 10 μM, 5–10 min each) failed to stimulate the FR of LC cells (effect of cirazoline 1 μM and 10 μM = −4.4% ± 1.4% and −7.9% ± 2.2%, respectively, *n* = 5 in both cases), whereas the highest concentration of the α_1_AR agonist (100 μM, 5–10 min) significantly stimulated the FR by 70.1% ± 22.6% (*n* = 5, *P* < 0.05) (Figures 2D,F). These results suggest that α_1_AR activation regulates the FR of LC neurons in a stimulatory way.

### 3.2 Molecular mechanisms involved in the α_1_AR-mediated effects of NA on the firing rate of LC cells

**FIGURE 3:**
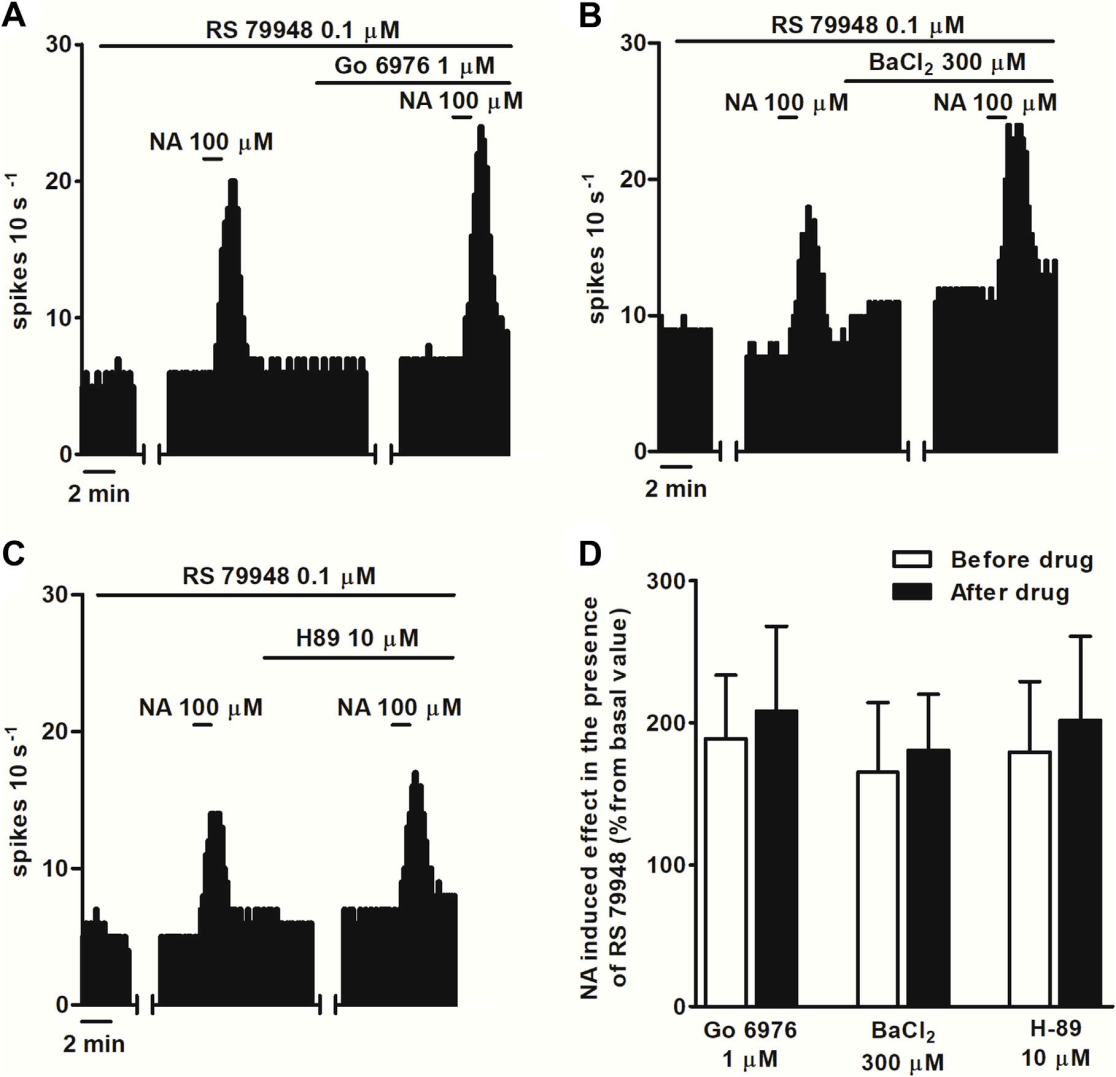
Effect of NA in the presence of RS 79948 before and after the application of PKC inhibitor Go 6976, GIRK blocker BaCl_2_, or PKA inhibitor H-89. **(A–C)** Representative examples of recordings of single LC neurons showing the stimulatory effect of NA (100 μM, 1 min) in the presence of RS79948 (0.1 μM, 10 min) before and after the application of Go 6976 (1 μM, 30 min) **(A)**, BaCl_2_ **(B)**, and H89 **(C)**. Vertical lines represent the integrated firing rates (spikes per 10 s). Drugs were bath-applied at the concentrations and for the durations indicated by the horizontal bars. **(D)** Bar histograms showing the mean ± SEM of the stimulatory effect (normalized as the percentage change from the baseline FR) of NA (100 μM, 1 min) in the presence of RS 79948 (0.1 μM, 10 min), before and after the application of Go 6976 (1 μM, 30 min, *n* = 5), BaCl_2_ (300 μM, 15 min, *n* = 5), or H-89 (10 μM, 20 min, *n* = 5).

**FIGURE 4:**
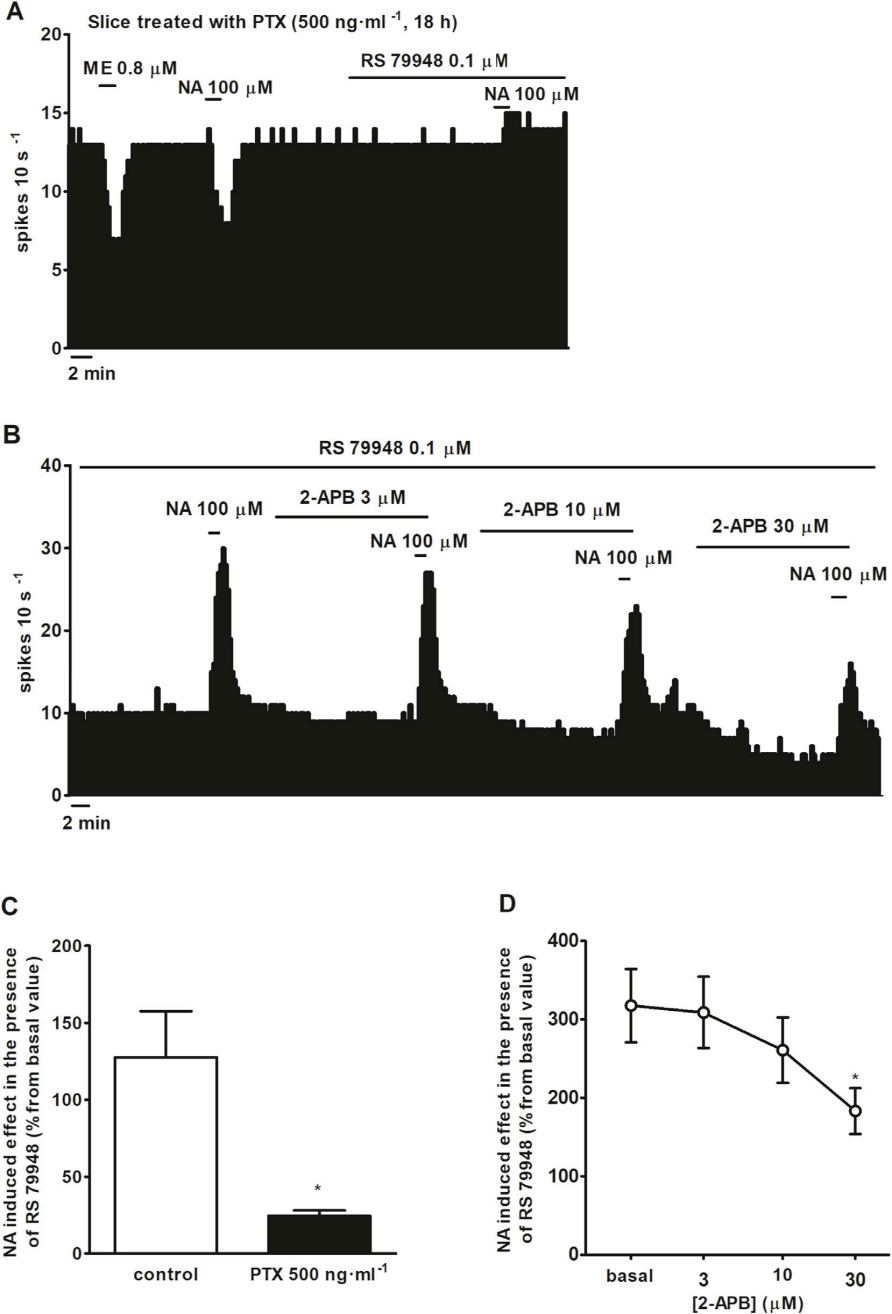
Effect of NA in the presence of RS 79948 before and after the application of the catalyst of ADP-ribosylation of G_i/o_ proteins PTX or the TRPC5/M7 channel blocker 2-APB. **(A,B)** Representative examples of the recordings of single LC neurons showing inhibitory effects of ME (0.8 μM, 1 min) and NA (100 μM, 1 min), and effect of NA (100 μM, 1 min) in the presence of RS 79948 (0.1 μM, 10 min), in a slice treated with PTX (500 ng ml^-1^, 18 h) **(A)**, or the effect of NA (100 μM, 1 min) in the presence of RS 79948 (0.1 μM, 10 min) before and after the application of 2-APB (3, 10, 30 μM, 10 min) **(B)**. Vertical lines represent the integrated firing rates (spikes per 10 s). Drugs (except PTX) were bath-applied at the concentrations and for the durations indicated by horizontal bars. **(C)** Bar histograms showing the mean ± SEM of the stimulatory effect of NA (100 μM, 1 min) in the presence of RS 79948 (0.1 μM, 10 min) in slices treated with PTX (500 ng ml^-1^, 18 h, *n* = 5) or its vehicle (*n* = 5). **(D)** Symbols representing the mean ± SEM of the stimulatory effect (normalized as the percentage change from the baseline FR) of NA (100 μM, 1 min) in the presence of RS 79948 (0.1 μM, 10 min), before and after the application of 2-APB (3, 10, and 30 μM; 10 min). *P < 0.05, compared with the stimulatory effect of NA (100 μM, 1 min) during RS 79948 (0.1 μM, 10 min) perfusion in slices that were not treated with PTX using an unpaired Student’s t-test. *P < 0.05, compared with the stimulatory effect of NA (100 μM, 1 min) during RS 79948 (0.1 μM, 10 min) perfusion before the application of 2-APB (3, 10, and 30 μM; 10 min) using a repeated-measures ANOVA, followed by Bonferroni’s multiple-comparison *post hoc* test.

To characterize which signaling pathways were involved in the stimulatory effect produced by NA after blockade of α_2_AR, we tested the effect of NA (100 μM, 1 min) in the continuous presence of RS 79948 (0.1 μM, 10 min), before and after the application of inhibitors of several signaling pathways that could be involved in the increase in the FR. Perfusion with the inhibitor of classical type PKC isoenzyme Go 6976 (1 μM, 30 min) (*n* = 5), PKA inhibitor H-89 (10 μM, 20 min) (*n* = 5), or GIRK channel blocker BaCl_2_ (300 μM, 15 min) (*n* = 5) failed to significantly change NA (100 μM, 1 min)-induced stimulation in the continuous presence of RS 79948 (0.1 μM, 10 min) (Figures 3A–D). To study the role of G_i/o_ proteins in the stimulatory effect induced by NA (100 μM, 1 min) in the presence of RS 79948 (0.1 μM, 10 min), we treated brain slices containing the LC with the catalyst of ADP-ribosylation of G_i/o_ proteins PTX (500 ng ml^-1^, 18 h). To assess whether G_i/o_ proteins had been correctly inactivated, we tested the inhibitory effect of μ opioid receptor agonist ME (0.8 μM, 1 min) in PTX-treated slices. μ opioid receptors couple to G_i/o_ proteins, and their activation causes the opening of GIRK channels, which, in turn, activate outward potassium currents that inhibit the FR of LC neurons (Stein, 2016). In PTX-treated slices, the inhibitory effect of ME (0.8 μM, 1 min) was significantly lower than that in control slices. Thus, ME (0.8 μM, 1 min)-induced inhibition in control slices was 96.7% ± 1.3% (*n* = 17), whereas in PTX-treated slices, it was 30.9% ± 7.6% (*n* = 5) (*P* < 0.05). This indicates that G_i/o_ protein inactivation by bath application of PTX (500 ng ml^-1^, 18 h) was effective. In slices treated with PTX (500 ng ml^-1^, 18 h), perfusion with NA (100 μM, 1 min) in the presence of RS 79948 (0.1 μM, 10 min) stimulated the FR by 29.4% ± 5.7% (*n* = 5, *P* < 0.05), which was significantly reduced compared to that in the control group (Figures 4A,C). Finally, we tested the effect of the TRPC5/M7 channel blocker 2-APB (3, 10, and 30 μM; 10 min) in the NA-induced stimulatory effect. Perfusion with 2-APB (3 and 10 μM, 10 min) leads to a reduction in the stimulatory effect induced by NA (100 μM, 1 min) in the presence of RS 79948 (0.1 μM, 10 min), which was significant with the highest concentration (30 μM, 10 min, *P* < 0.05) (Figures 4B,D). Thus, before 2-APB perfusion, the stimulatory effect of NA (100 μM, 1 min) in the presence of RS 79948 (0.1 μM, 10 min) was 449.2% ± 97.7%, whereas after 2-APB perfusion (3 μM, 10 μM, and 30 μM; 10 min), it was 434.8 ± 91.0 (*n* = 5), 363.6% ± 73.9% (*n* = 5), and 253.8% ± 49.4% (*n* = 5, *P* < 0.05), respectively (Figures 4B,D). These data suggest that both G_i/o_ proteins and transient receptor potential (TRP) channels are implicated in the signaling pathway that mediates the stimulatory effect induced by NA (100 μM, 1 min) in the presence of RS 79948 (0.1 μM, 10 min) through α_1_AR.

### 3.3 Molecular mechanisms involved in the α_1_AR-mediated effect of cirazoline

**FIGURE 5:**
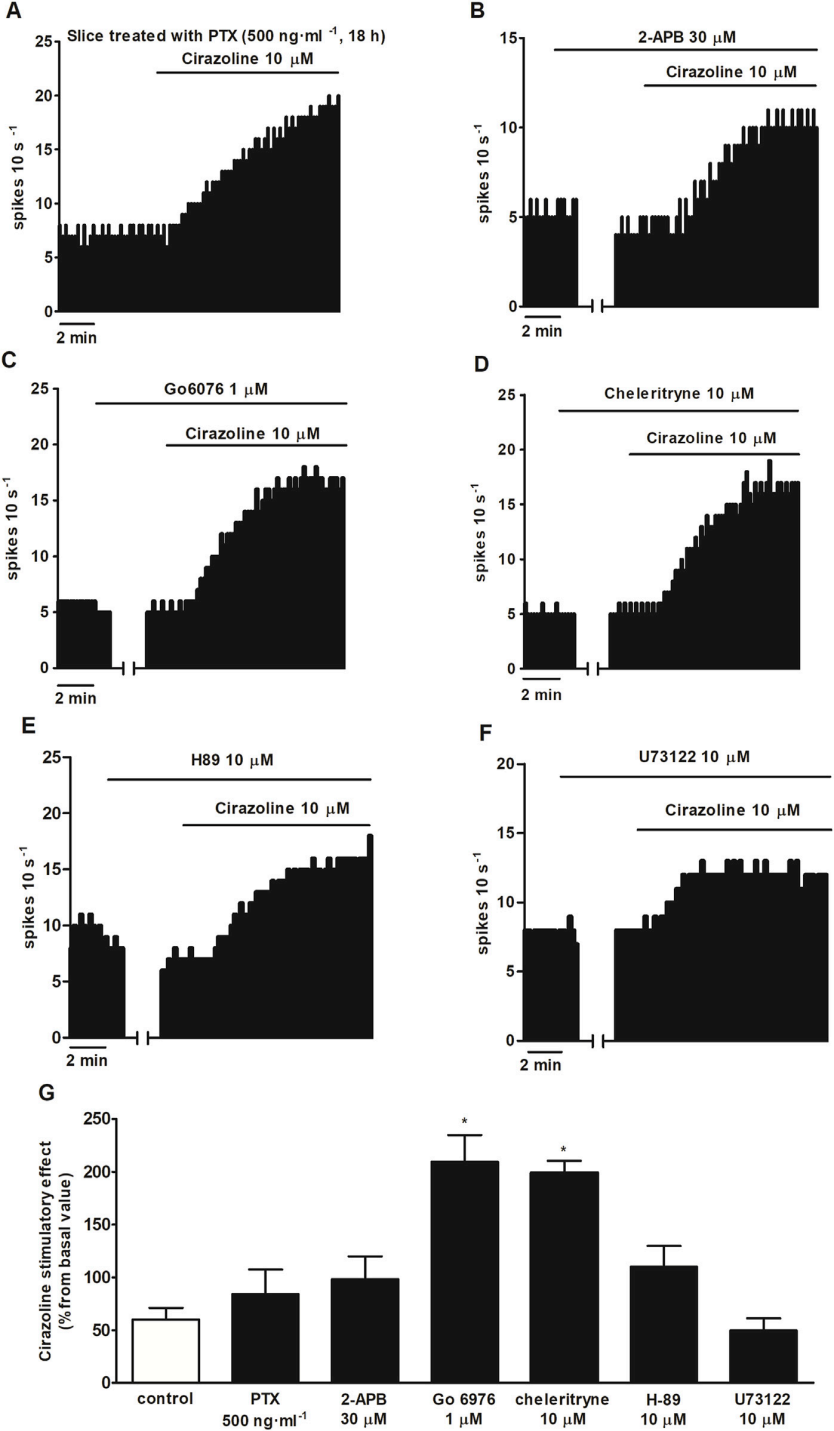
Effect of cirazoline in the absence or presence of PTX, 2-APB, Go 6976, chelerythrine, H-89, and U73122. **(A,B)** Representative examples of the recordings of single LC neurons showing the stimulatory effect of cirazoline (10 μM, 5–10 min) in a slice treated with PTX (500 ng ml^-1^, 18 h) **(A)** or in the presence of 2-APB (30 μM, 10 min) **(B)**, Go 6976 (1 μM, 30 min) **(C)**, chelerythrine (10 μM, 30 min) **(D)**, H-89 (10 μM, 20 min) **(E)**, or U73122 (10 μM, 30 min) **(F)**. Vertical lines represent the integrated firing rates (spikes per 10 s). Drugs (except PTX) were bath-applied at the concentrations and for the durations indicated by horizontal bars. **(G)** Bar histograms showing the mean ± SEM of the stimulatory effect of cirazoline (normalized as the percentage change from the baseline FR) in the absence (control) and presence of PTX (500 ng ml^-1^, 18 h), 2-APB (30 μM, 10 min), Go 6976 (1 μM, 30 min), chelerythrine (10 μM, 30 min), H-89 (10 μM, 20 min), and U73122 (10 μM, 30 min). *P < 0.05, compared with the stimulatory effect of cirazoline (10 μM, 5–10 min) in the presence of the vehicle using one-way ANOVA, followed by the Dunnett *post hoc* test.

To further study the mechanism of the stimulatory effect mediated by α_1_AR activation, we tested the effect of α_1_AR agonist cirazoline (10 μM, 5–10 min) after treatment with inhibitors of several signaling pathways. As previously mentioned, the cirazoline (10 μM, 5–10 min)-induced effect was 60.1% ± 11.2% (*n* = 12, *P* < 0.05). Unexpectedly, application of the PKC inhibitors Go 6976 (1 μM, 30 min) and chelerythrine (10 μM, 30 min) induced a significant increase in cirazoline (10 μM, 5–10 min) stimulatory effect (cirazoline’s effect after Go 6976 = 209.3 ± 25.6%, *n* = 5; after chelerythrine = 199.2 ± 11.1%, *n* = 3; *P* < 0.05 in both cases) (Figures 5C,D,G). However, slice treatment with the G_i/o_ protein inactivator PTX (500 ng ml^-1^, 18 h) (*n* = 5), the blocker of TRPC5 and TRPM7 subtypes 2-APB (30 μM, 10 min) (*n* = 5), the PKA inhibitor H-89 (10 μM, 20 min (*n* = 5), or the PLC inhibitor U73122 (10 μM, 30 min) (*n* = 6) failed to change cirazoline (10 μM, 5–10 min)-induced stimulation (Figures 5A,B,E–G). These results suggest that the signaling pathways that were studied are not directly involved in the stimulatory effect induced by cirazoline through the α_1_AR.

## 4 Discussion

The present work was undertaken to investigate the role of the α_1_AR in the regulation of the FR of LC neurons and the signaling pathways involved in its effects. Our results reveal that α_1_AR activation with the adrenergic agonists NA and PE in the presence of an α_2_AR antagonist or with cirazoline stimulates the FR of LC NA cells in adult male rat brain *ex vivo.* NA-induced stimulation was reduced by the inhibitor of G_i/o_ protein PTX and the TRP channel blocker 2-APB. However, none of the inhibitors blocked the stimulatory effect induced by cirazoline.

Perfusion with NA or PE inhibits the FR of LC neurons, whereas in the presence of α_2_AR antagonist RS 79948, they stimulate the FR. These results show that the inhibitory effects of these adrenergic agonists are mediated by the α_2_AR, as previously described (Williams et al., 1985; Williams et al., 1991). Even though PE is mainly considered a selective α_1_AR agonist, some studies performed in vascular tissues have shown that PE can activate α_2_AR (McGrath et al., 1999; Görnemann et al., 2009; VanLangen et al., 2013). Moreover, PE partially inhibits serotonin release in rat raphe nuclei through α_2A_AR activation (Hopwood and Stamford, 2001) and also evokes small-membrane hyperpolarizations in LC neurons (Williams et al., 1985). Finally, it has been recently reported that PE stimulates the cytoplasmatic release of NA via the NA transporter (Al-Khrasani et al., 2022), which could explain the inhibitory effect of PE observed in our experiments due to the presence of large reserve of α_2_AR receptors in LC neurons (Pineda et al., 1997).

In the presence of RS 79948 and α_1_AR antagonist WB 4101, both the inhibitory and stimulatory effects of NA and PE were blocked, which indicates that the increase in the FR induced by NA or PE after the α_2_AR blockade is mediated by the α_1_AR. Our data suggest that the α_1_AR-mediated excitatory effect of NA in LC neurons in adult rats may be masked by concurrent α_2_AR-mediated inhibition becoming apparent only when α_2_ARs are blocked by an antagonist. These results are consistent with those of studies that suggest that the activation of the α_1_AR may contribute to increase the FR of LC neurons (Ivanov and Aston-Jones, 1995). In contrast, some previous studies have reported that α_1_AR-mediated effects in LC neurons are restricted to early developmental stages. This discrepancy may be explained, at least in part, through methodological differences. Notably, earlier studies used techniques such as intracellular recordings in organotypic cultures, which may not fully reflect the physiological properties of mature LC neurons in acute brain slices. Moreover, the studies did not examine the effects of cirazoline or assess NA responses in the presence of an α_2_AR antagonist, both key aspects of our experimental design that may have unmasked α_1_AR-mediated excitatory effects in adult tissue.

The putative involvement of the β_1_AR in the stimulatory effects induced by NA and PE could be considered as both compounds bind to this receptor subtype (Ki ≈ 100–300 nM and 13 μM, respectively). However, in the case of NA, the contribution of the β_1_AR appears unlikely as WB 4101—a selective α_1_AR antagonist that does not bind to β_1_AR—completely blocked the stimulatory effect of NA in the presence of α_2_AR antagonist RS 79948. In contrast, in our slice preparations, WB 4101 significantly, but not completely, inhibited the stimulatory effect of PE under the same conditions. This suggests that β_1_AR may contribute to the effects of PE, a possibility that cannot be entirely excluded considering their affinity values for different receptors (Ki ≈ 13 µM for β_1_AR, 6 µM for α_1_AR, and 0.4 µM for α_2_AR) and the concentration applied (Chen et al., 1993; Gil and Donello, 2005). Alternatively, the involvement of other receptors (e.g., 5-HT_7_ receptor) could not be ruled out (European Molecular Biology Laboratory - European Bioinformatics Institute, 2025). It is important to note, however, that the same concentration of PE (100 µM) has previously been administrated in LC slice preparations to investigate α_1_AR-mediated effects, which aligns with the conditions used in our study (Osborne et al., 2002). Furthermore, the concentrations of all drugs used in this study were selected based on their reported Ki values or data from previous electrophysiological studies in brain slices, taking into account that drug affinities observed in isolated radioligand binding assays can differ significantly—often by 10- to 300-fold depending on the drug hydrosolubility—from those used in functionally active slice preparation. We used antagonists RS 79948 and WB 4101 to block α_2_AR and α_1_AR, respectively, because they have been shown to be rather selective for each α-adrenergic receptor type in previous *in vivo* or binding studies (Drew, 1982; Michel et al., 1995; Milligan et al., 1997; Mateo et al., 2000; Proudman et al., 2022). In other words, WB 4101 has lower affinity for D_2_ (Ki ≈ 123 nM) and α_2_AR (Ki ≈ 28–46 nM for α_2B_–α_2A_AR) than for α_1_AR (Ki ≈ 6–8 nM for α_1B_ and Ki ≈ 0.5 nM for α_1A_). We did not use prazosin or terazosin as antagonists due to their lower selectivity for the α_1A_AR (Hancock et al., 1995; Yuan et al., 2009). Furthermore, WB 4101 (0.5 µM) blocks the PE-induced effect through the α_1A_AR in slices from other brain regions (Hancock et al., 1995; Yuan et al., 2009), which could also occur in the LC.

The α_1_AR agonist cirazoline, which also shows moderate affinity for the 5-HT_1A_ receptor (Ki ≈ 35 nM) and binds to the α_2_AR (Ki ≈ 59 nM), stimulated the FR of LC cells. The stimulatory effects induced by cirazoline (1 µM and 10 µM) were blocked by perfusion with α_1_AR antagonist WB 4101, supporting the role of the α_1_AR in the regulation of the FR of LC neurons. However, at high concentrations (100 µM), cirazoline-induced stimulation was not blocked by WB 4101, which indicates that WB 4101 behaves as a competitive antagonist. The involvement of the non-α_1_AR receptor-mediated mechanism in the effect produced by the highest concentration of cirazoline could also be considered, including the activation of imidazoline receptors, 5-HT_1A_ receptors, and α_2A_AR (Angel et al., 1995; Alexander et al., 2023). Thus, in anesthetized rats pretreated with EEDQ (an irreversible α-adrenoceptor antagonist), imidazoline drugs such as clonidine, cirazoline, and rilmenidine stimulate neuronal activity in LC cells through the activation of I_1_-imidazoline receptors (Pineda et al., 1993), but it seems to be an indirect effect mediated by imidazoline receptors located on paragigantocellularis neurons that project to the LC (Ruiz-Ortega and Ugedo, 1997). Some imidazoline drugs can stimulate the FR of LC neurons by a non-I_1_/I_2_ imidazoline receptor located extracellularly (Ugedo et al., 1998). Although this mechanism has not been described for cirazoline, it remains to be studied how cirazoline stimulates the FR of LC neurons after blockade of the α_1_AR in our system. The putative contribution of the 5-HT_1A_ receptor or α_2_AR to the observed stimulatory effect could be ruled out as the activation of the 5-HT_1A_ receptor or α_2_AR would reduce rather than increase the FR of LC cells. To address the study of the signaling pathways involved in the stimulatory effect mediated by α_1_AR, we used the agonists NA (100 μM; in the presence of the α_2_AR antagonist RS 79948 0.1 µM) and cirazoline (10 µM) because their effects were fully blocked after α_1_AR antagonism. Even though α_1_AR has been considered to be coupled to the G_q/11_/PLC/PKC pathway in some areas of the rat brain (Kobayashi et al., 2008), there is evidence showing that this receptor can couple to other G proteins and multiple signaling pathways (Hein and Michel, 2007; Cotecchia, 2010). α_1_AR activation can also induce cAMP accumulation and PKA activation, which could modulate PKC (García-Sáinz et al., 2000). The enhancement of intracellular levels of cAMP or application of its analogs is known to increase the FR of LC neurons (Wang and Aghajanian, 1987). The activation of the α_1_AR with PE suppresses currents carried by GIRK channels that are opened by Gi/o protein-coupled receptors such as µ opioid receptor or α_2_AR (Osborne et al., 2002). In addition, it has been described that TRP channel antagonists suppress inward currents produced after α_1_AR activation with phenylephrine in LC neurons of SHR juvenile rats (Igata et al., 2014). Moreover, quantitative real-time PCR analysis found high levels of mRNA expression of TRPC5 (the most abundant type), TRPM7, and TRPM2 channels in the LC (Cui et al., 2011). The drugs that were used to inhibit each signaling pathway, such as Go 6976, PTX, BaCl_2_, 2-APB, H-89, and U73122, had been previously used by other authors in rat brain slices at similar concentrations (Chiu et al., 1995; Chessel et al., 1996; Bailey et al., 2009; Murai et al., 2012; Igata et al., 2014; Jolas et al., 2000).

PKC inhibitor Go 6976, PKA inhibitor H-89, and the blockade of GIRK channels with BaCl_2_ failed to change the stimulatory effect of NA in the presence of RS 79948. In contrast, G_i/o_ protein inhibition with PTX and perfusion with the TRPC5 and TRPM7 channel blocker 2-APB significantly reduced the stimulatory effect of NA in the presence of RS 79948, suggesting that the effect induced by NA through α_1_AR occurs via a pathway that involves G_i/o_ proteins and TRP channels. Although there is no evidence describing the α_1_AR/G_i/o_ protein/TRP channel pathway in neurons, some studies performed in different tissues could support this hypothesis. First, α_1_AR couples to G_i/o_ proteins and mediates pertussis toxin-sensitive effects in some vascular systems (Gurdal et al., 1997; Otani et al., 2001; Petitcolin et al., 2001). Second, G_i/o_ proteins stimulate TRPC5 and TRPM7 channel activities in several cell types (Beech, 2012; Oronowicz et al., 2021). Moreover, TRPC5 channels can be activated by the G_i/o_ protein-coupled µ opioid receptor (Miller et al., 2011), which is widely expressed in LC neurons, and they also contribute to the development of opioid tolerance in spinal neurons (Chu et al., 2020). Considering the aforementioned studies, we suggest that α_1_AR activation by adrenergic agonist NA stimulates the FR of LC neurons through its interaction with G_i/o_ proteins and TRPC5/TRPM7 channels, and we propose two hypotheses that could explain this mechanism. On the one hand, α_1_AR, G_i/o_ proteins, and TRP channels could be directly coupled. Then, α_1_AR activation could regulate G_i/o_ proteins and lead to the opening of TRP channels, which would induce an inward cationic current to increase the FR. On the other hand, Gi/o proteins could constitutively inhibit TRP channels and α_1_AR activation could relieve this inhibition, which would result in TRP channel opening and neuron depolarization.

It has been described that α_1_AR activation by PE in LC neurons reduces GIRK channel conductance induced by α_2_AR or µ opioid receptors, which are coupled to G_i/o_ proteins (Osborne et al., 2002). This interaction does not seem to occur in our system as the role of GIRK channels in α_1_AR activation was discarded. However, a mechanism involving TRP channels could also explain the observed stimulatory effect of PE on the FR of LC cells as in the case of NA. In contrast to NA-induced stimulation, G_i/o_ protein inhibitor PTX, TRP channel blocker 2-APB, PKC inhibitors Go 6976 and chelerythrine, PKA inhibitor H89, and PLC inhibitor U73122 failed to reduce the cirazoline stimulatory effect.

Furthermore, Go 6976 and chelerythrine enhanced the effect of cirazoline. Although no studies so far have directly linked PKC inhibition to the increased cirazoline effect, it is plausible to hypothesize that PKC activity exerts a constitutive inhibitory influence on the signaling pathway responsible for α_1_AR-mediated increases in the firing rate of LC neurons. Therefore, inhibition of PKC may relieve this suppression, thereby amplifying the stimulatory effect of cirazoline.

The differences in the results regarding the signaling pathways involved in NA or cirazoline stimulatory effects can be explained because these agonists may display functional selectivity. Functional selectivity or biased signaling refers to the ligand-dependent receptor activation of certain signaling pathways over others (Kolb et al., 2022). This property has been widely characterized in many GPCRs, including α_1_AR, which has been described by several authors to couple to different G proteins and activate several signaling pathways (Zhong and Minneman, 1999; Alcántara-Hernández et al., 2017; da Silva Junior et al., 2017). Therefore, our results could be explained due to biased signaling as studies performed in CHO-K1 cells expressing α_1a_AR found that cirazoline displays the signaling bias toward cAMP accumulation relative to Ca^2+^ release when compared to reference endogenous agonist NA (Evans et al., 2011). Moreover, NA and cirazoline show different affinities for each α_1_AR receptor subtype (NA: α_1d_ > α_1b_ > α_1a_; cirazoline: α_1a_ > α_1d_ > α_1b_) (Horie et al., 1995; Proudman and Baker, 2021).

Our study has some potential limitations. First, the magnitude of the basal stimulatory effect of NA in the presence of RS 79948 (i.e., in the absence of inhibitors or blockers) appeared to differ across some experimental groups (e.g., Figures 1 vs*.* Figure 4). However, no changes in experimental conditions could account for this discrepancy. Notably, in most groups, the effect of NA in the presence of RS 79948 was compared to the control NA response recorded in the same neuron, minimizing the impact of this variability on the overall conclusions. Second, consistent with previous studies, only male rats were used in this investigation. Future experiments should aim to characterize α_1_AR-mediated effects in female rats to assess potential sex-dependent differences.

α_1_AR is involved in several CNS functions and pathologies such as behavioral activity and depression (Stone et al., 2007), pain modulation (Kingery et al., 2002; Nakatsuka et al., 2022), reward processes (Lin et al., 2007), psychostimulant-induced locomotor hyperactivity (Drouin et al., 2002), or neurodegenerative disorders. Some of these have been shown to be related to the LC. In this line, an increase in the neuronal activity induced by the activation of α_1_-AR has also been reported in other brain regions, such as in the prefrontal cortex (Datta et al., 2019), the paraventricular nucleus of the hypothalamus (Chen et al., 2006), or the interneurons of the layer CA1 of the hippocampus (Hillman et al., 2009). In the latter case, this activation leads to a decrease in the activity of pyramidal neurons and to an antiepileptic effect. Previous studies have revealed a role of α_2_AR in the regulation of the activity of LC neuron (Aghajanian and VanderMaelen, 1982; Pineda et al., 1997) in the adult rat brain, but the function of α_1_AR was thought to be reduced during development. Our results reveal a functional importance of α_1_AR in the adult rat LC. The α_1_AR can be activated by adrenergic agonists NA and PE (after α_2_AR blockade) or by α_1_AR agonist cirazoline, stimulating the FR of LC neurons. The stimulatory effect induced by NA would occur through a signaling pathway that involves G_i/o_ proteins and TRPC5/TRPM7 channels. More studies will be required to describe in detail the mechanisms involved in the α_1_AR stimulatory effect and the functional role of this receptor in female rats, but our results suggest that the α_1_AR receptor in the adult male rat LC could constitute a target in the treatment of several disorders of the CNS.

## Data Availability

The raw data supporting the conclusions of this article will be made available by the authors, without undue reservation.

## Abbreviations

α_1_AR, α_1_-adrenoceptor; α_2_AR, α_2_-adrenoceptor; aCSF, artificial cerebrospinal fluid; βAR, β-adrenoceptor; CNS, central nervous system; cAMP, cyclic adenosine monophosphate; D_2_ receptor, dopamine D_2_ receptor; FR, firing rate; GABA, γ-aminobutyric acid; GIRK, G protein-activated inward rectifier potassium; GPCR, G protein-coupled receptor; 5-HT_1A_ receptor, serotonin 1A receptor; LC, locus coeruleus; ME, [Met]enkephalin; NA, noradrenaline; PAG, periaqueductal gray; PE, phenylephrine; PKA, protein kinase A; PKC, protein kinase C; PLC, phospholipase C; PTX, pertussis toxin; TRP, transient receptor potential channel.
